# A sustainable mixed diet for children without compromising nutritional needs: The vitamin B12 issue

**DOI:** 10.1002/fsn3.4491

**Published:** 2025-01-28

**Authors:** Hermann Kalhoff, Mathilde Kersting, Kathrin Sinningen, Thomas Lücke

**Affiliations:** ^1^ Research Department of Child Nutrition, University Hospital of Pediatrics and Adolescent Medicine, St. Josef‐Hospital Ruhr‐University Bochum Bochum Germany

**Keywords:** children, mixed diets, planetary health, sustainability, vitamin B12

## Abstract

Global climate change requires a paradigm change in diets, especially in affluent countries, with a reduction of animal‐based food, including children. This will have direct consequences for Vitamin B12 supply as animal‐based foods are the only food source. We examined these potential consequences using the German food based dietary guidelines for infants, children, and adults in Germany as the basis for the model calculation. The guidelines for 4 representative age groups were examined, representing exclusive milk feeding (2 months), complementary feeding (8 months), and mixed family diet (children: 4–6 years, adolescents: 11–14 years). For each age group (except 2 months), the contribution of animal‐based food groups (milk/dairy, meat, fish, eggs) to the total daily intake of vitamin B12 was calculated based on 7‐day menus with recipes for all meals. This allowed us to assess the potential Vitamin B12 deficits due to food group exclusion. Even in the guidelines diets, including exclusive breastfeeding, the vitamin B12 intakes just reached the reference values in all age groups. In infants on complementary feeding and also later in children and adolescents, cow's milk was by far the most important source of vitamin B12. Among the other animal‐based foods, meat (16.9%–23.0%) ranked first, followed by fish (11.0%–16.5%), and eggs (8.1%). In our analysis of the German food‐based guidelines for infant, child, and adolescent diets, the increased planetary health due to reduction of milk intake turned out severely to compromise vitamin B12 intake. In children, a reduction in the consumption of animal foods to improve the health of the planet must be weighed against the risk of inadequate intake of individual nutrients.

## Graphical abstract

Global climate change requires a paradigm shift in diet, especially in affluent countries with a reduction in animal foods. Our analysis shows that the improvement in sustainability comes at the cost of a reduced intake of B12 due to the exclusion of milk from children's diets. Sustainable nutritional recommendations must therefore seek a balance between planetary health and adequate nutrient supply for children's growth.

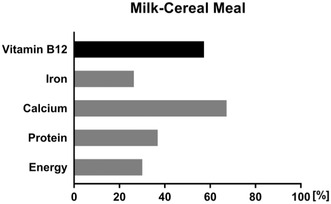

## 1 INTRODUCTION

In the human diet, animal‐based food is the only natural source of active Vitamin B12 (also called cobalamin) (Obeid et al., 2019). Because of the specific roles of Vitamin B12 in cellular metabolism, including DNA synthesis, methylation, and mitochondrial metabolism, infants, children, adolescents, and women of childbearing age are among the groups with a high risk of insufficiency if consumption of animal‐based food is restricted (Green et al., 2017; Stabler, 2013). Except as a Vitamin B12 source, animal‐based foods are especially important as highly relevant sources of individual nutrients in the growth phase, such as milk for calcium and Vitamin B2, meat for iron and zinc, and fish for iodine and fatty acids, and each of them for high‐quality protein.

In recent years, the definition of a healthy diet has been expanded beyond the pure need for nutrients in order to simultaneously ensure the sustainability of food systems (Eat Lancet Commission, 2019). As a consequence, plant‐based diets come into focus, meaning a reduced consumption of animal‐based foods in the western world. Depending on the rigor of the reduction of animal foods, one ends up with vegetarian and vegan diets, which may also be chosen for ethical reasons (Kersting et al., 2018).

To evaluate putative health risks from reducing some or all animal‐based foods from the diet, for instance, to derive realistic public health messages or to provide dietary counseling to families, precise and comprehensive information on the usual daily food and nutrient intake of infants, children, and adolescents is needed. As such precise data is often not feasible in everyday life, quantitative guidelines for daily food intake are an advantage. In Germany, such food‐based dietary guidelines for infants, children and adolescents have existed for many years and are continuously reviewed to assure adequate nutrient intakes in accordance with reference values (Kersting et al., 2005, 2017, 2020).

The aim of this study was to evaluate the specific contribution of the different animal‐based food groups in a nutritionally balanced diet to vitamin B12 sufficiency throughout the whole growth period.

## 2 MATERIALS AND METHODS

This is a theoretical model calculation. The amounts of food consumed for the various age groups were deduced from the menus that form the basis of the national dietary recommendations.

### 2.1 Quantitative food‐based guidelines

**FIGURE 1:**
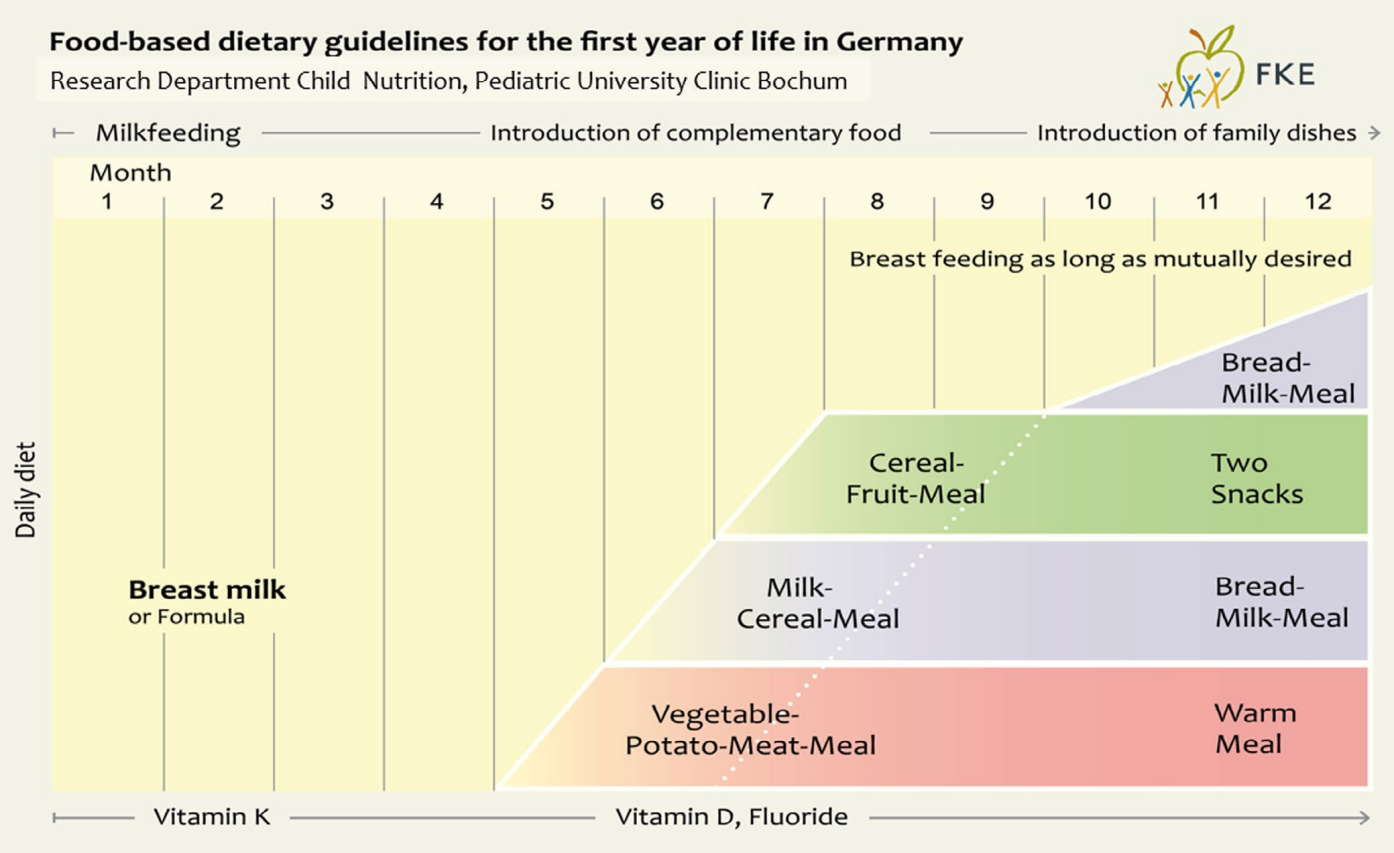
Dietary scheme for the first year of life. Research Department of Child Nutrition (FKE), Bochum, Germany.

**FIGURE 2:**
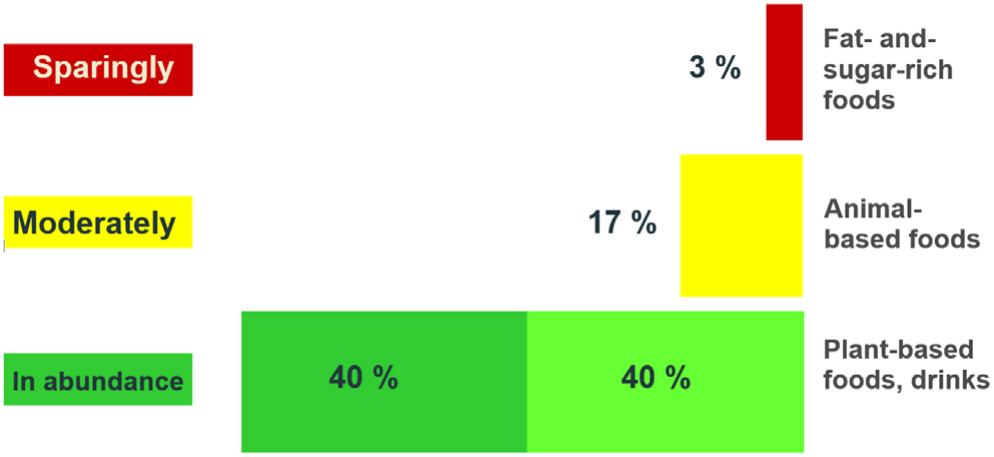
Optimized mixed diet for children and adolescents. Research Department of Child Nutrition (FKE), Bochum, Germany.

The German food‐based dietary guidelines, namely the “Nutritional Scheme” for the first year of life (Kersting et al., 2020) (Figure 1) and the “Optimized Mixed Diet” for children and adolescents aged 1–17 years (Kersting et al., 2005, 2017) (Figure 2), are each based on 7‐day menus with recipes for all meals and reflect German dietary culture and habits.

### 2.2 First year of life

In the first year of life, three developmental phases that gradually merge into one another can be distinguished (Kersting et al., 2020): exclusive breastfeeding during the first months, introduction of complementary feeding including whole cow's milk along with partial breastfeeding starting between 4 and 6 months, and introduction of the Optimized Mixed Diet as family diet toward the end of the first year of life. Infant formula or follow‐on formula is used as a breastmilk substitute, depending on age. Except formula, no other fortified foods were used in the recommended diets at any age (Figure 1).

Food amounts for exclusive milk feeding were based on the median consumption of infant formula within the first 16 weeks of life (boys 846 mL/day) derived by the European Food Safety Authority (EFSA, 2017). For breastfeeding, these values were reduced by 10% because the few available studies that measured the breast milk consumption point to slightly lower amounts compared to formula.

The food amounts of the mixed diet (8 months of age) were based on a 7‐day sample menu with 3 solid complementary meals + fluid milk in infancy. The recipes of the complementary meals refer to the age of 8 months, with meat (30 g) five times per week, fish (30 g) once per week, and whole cow's milk (200 mL) daily in a milk–cereal meal (Table 1).

**TABLE 1:** Dietary scheme for the first year of life: Typical day of the 7‐day menu (animal‐based food groups in capital letters).

Foods / meals	Typical day of the 7 day menu
Breastmik/Formula	200 g
Vegetable‐potato‐meat meal	
Vegetables	100 g fennel
Potato, Pasta, Rice	50 g rice
MEAT, FISH	30 g POULTRY
Rapeseed oil	5 g
Milk‐–Cereal meal	
COW'S MILK	200 g
Cereal, wholegrain	20 g wheat
Orange juice	20 g
Cereal–Fruit Meal	
Cereal, wholegrain	20 g oats
Fruit	100 g pear
Water	90 g
Rapeseed oil	5 g

### 2.3 Children and adolescents

The main message of the Optimized Mixed Diet can be summarized in 3 simple rules for food selection (see Figure 2); one of them is that foods of animal origin should be consumed in moderate amounts.

The food amounts of the mixed diet were based on a 7‐day sample menu of 3 main meals + 2 snacks for children and adolescents (Table 2). The recipes of the Optimized Mixed Diet refer to children aged 4–6 years (exemplary sample group), with average daily amounts of about 330 mL dairy (calculated as fluid cow's milk), 30 g meat, 10 g fish, and 10 g eggs.

**TABLE 2:** Optimized mixed diet for children and adolescents, calculated for the 4–6 year‐old reference age group: Typical day of the 7‐day menu (animal‐based food groups in capital letters).

Breakfast	Morning snack	Lunch	Afternoon snack	Dinner
*Bircher muesli*	*Sausage bread with fruits*	*Potato‐millet pan*	*Fruits with chocolate*	*Potato salad*
COW'S MILK 100 mL	Grapes 50 g	Potatoes 120 g	Banana 30 g	Potatoes 120 g
Apple 60 g	Wohle grain bread 40 g	Carrots 35 g	Pineapple 30 g	Cucumber 100 g
Oat flakes 40 g	BOLOGNA SAUSAGE 25 g	Peas 30 g	Strawberry 30 g	Onions 20 g
Cornflakes 20 g	Tomatoes paste 5 g	COW'S MILK 20 mL	Chocolate 20 g	Rapeseed oil 18 g
Nuts grounded 15 g		Millet 15 g		Vinegar 10 g
Raisins 7 g		Gouda 8 g		
		EGG 7 g		
		Leeks 5 g		
		Rapeseed oil 4 g		
		Margarine 4 g		
		Breadcrumbs 3 g		
Water 150 mL	Water 200 mL	Water 150 mL	Water 200 mL	Water 200 mL

For the other age groups, the food proportions (and their shares of nutrient intake) are identical, but the daily amounts differ according to specific age‐dependent energy needs (Kersting et al., 2005, 2017). This principle enabled a quantitative assessment of animal food intake in our fourth age group (adolescents aged 11–14 years).

### 2.4 Vitamin B12 intake

#### 2.4.1 Exclusive milk feeding

Data on nutrient content in mature human milk was taken from the German standard nutrient tables (Souci et al., 2018). Data on nutrient content in formula was taken as average values from a recent market survey in Germany covering 37 infant formula products (exclusive milk feeding period) and 17 follow‐on formula products (complementary feeding period).

#### 2.4.2 Mixed diets

To calculate the nutrient intake per meal and per day, nutrition analysis software common in Germany (DIAT‐2020 Soft & Hard, D. Beyer, Rimbach, Germany) was used. Nutrient values were obtained from the German Food Code and Nutrient Database (BLS, Bundeslebensmittelschluessel, BLS, Version II.3.1), which has already been used repeatedly for nutrient intake calculation at the European level in the HELENA study (Wisnuwardani et al., 2019). The BLS considers nutrient losses because of food preparation, such as cooking vegetables or meat.

As a target for Vitamin B12 intake with the guideline diets, reference values from the EFSA and more recent values from the German, Austrian, and Swiss authorities (DACH) were used, defined as Adequate Intake (AI). Both institutions derived the values for the pediatric age groups above 4 months from their values for adults (DACH, 2018; EFSA, 2015). For the exclusive milk feeding period, DACH values are based on recent Vit B12 analyses in breastmilk (DACH, 2019) and EFSA values were taken from a recent summary (EFSA, 2015).

To assess the contribution of the different daily meals to the total nutrient intake, also protein, calcium, and iron were considered besides Vitamin B12 because animal‐based foods are relevant sources for these nutrients in child nutrition as well.

## 3 RESULTS

Vitamin B12 intake with the recommended diets increased from 0.40 μg/d in exclusively breastfed infants to about 4 μg/day in adolescents (Table 3). Reference values for Vitamin B12 intake were achieved in all age groups, but to varying degrees.

**TABLE 3:** Mean daily food group consumption and Vitamin B12 intake with the “Dietary Scheme for the first year of life” and the “Optimized Mixed Diet” for children and adolescents; reference values of EFSA and DACH for comparison.

	2 months breastmilk	2 months formula	8 months (with breastmilk)	8 months (with formula)	4–6 years	11–14 years girls^1^	11–14 years boys^1^
Breastmilk	800 g **0.40 μg** B_12_ [100%]		200 g **0.10 μg** B_12_ [7.1%]				
Formula		850 g **1.28 μg** B_12_ [100%]		200 g **0.30 μg** B_12_ [18.8%]			
Cow's milk/milk products^2^			200 g **0.80 μg** B_12_ [57.3%]	200 g **0.80 μg** B_12_ [50.1%]	363 g^**2**^ **1.38 μg** B_12_ [58.5%]	598 g^**2**^ **2.28 μg** B_12_ [58.5%]	613 g^**2**^ **2.34 μg** B_12_ [58.5%]
Meat			21 g **0.32** μg B_12_ [23.0%]	21 g **0.32 μg** B_12_ [20.1%]	27 g **0.40 μg** B_12_ [16.9%]	45 g **0.66 μg** B_12_ [16.9%]	46 g **0.67 μg** B_12_ [16.9%]
Fish			4 g **0.18** μg B_12_ [12.6%]	4 g **0.18 μg** B_12_ [11.0%]	10 g **0.39 μg** B_12_ [16.5%]	16 g **0.64 μg** B_12_ [16.5%]	17 g **0.66 μg** B_12_ [16.5%]
Eggs					10 g **0.19 μg** B_12_ [8.1%]	17 g **0.32 μg** B_12_ [8.1%]	17 g **0.33 μg** B_12_ [8.1%]
Vit B12 intake (μg/day)	**0.40**	**1.28**	**1.40**	**1.60**	**2.36**	**3.90**	**4,00**
EFSA (μg/day)	**0.4**	**0.4**	**1.5**	**1.5**	**1.5**	**3.5**	**3.5**
DACH (μg/day)	**0.5**	**0.5**	**1.4**	**1.4**	**2.0**	**3.5** ^**3**^	**3.5** ^**3**^

^1^ amounts differ according to age specific energy needs.

^2^ Milk equivalents, i.e. 100 g milk corresponds to 100 g yoghurt or 30 g cheese.

Bold values denote statistical significance at the calculated Vitamin B12 intake.

### 3.1 Vitamin B12 intake in infants

With exclusive breastfeeding and in the complementary feeding period, infants just reached the reference range, while intakes in children and adolescents were significantly above the references. With exclusive formula feeding, Vitamin B12 intake was three times higher than the reference values.

In the complementary feeding period, the milk‐related differences due to fortification of formula disappeared, as vitamin B12 intake is increasingly determined by common animal‐based complementary foods, mainly cow's milk in the milk–cereal meal.

### 3.2 Vitamin B12 intake in children and adolescents

With transition to the Optimized Mixed Diet, cow's milk (products) remained the most important Vitamin B12 source with about 60% of total B12 intake, while meat and fish made up 17% each and eggs 8% (Table 3).

### 3.3 Vitamin B12 intake with meals

**FIGURE 3:**
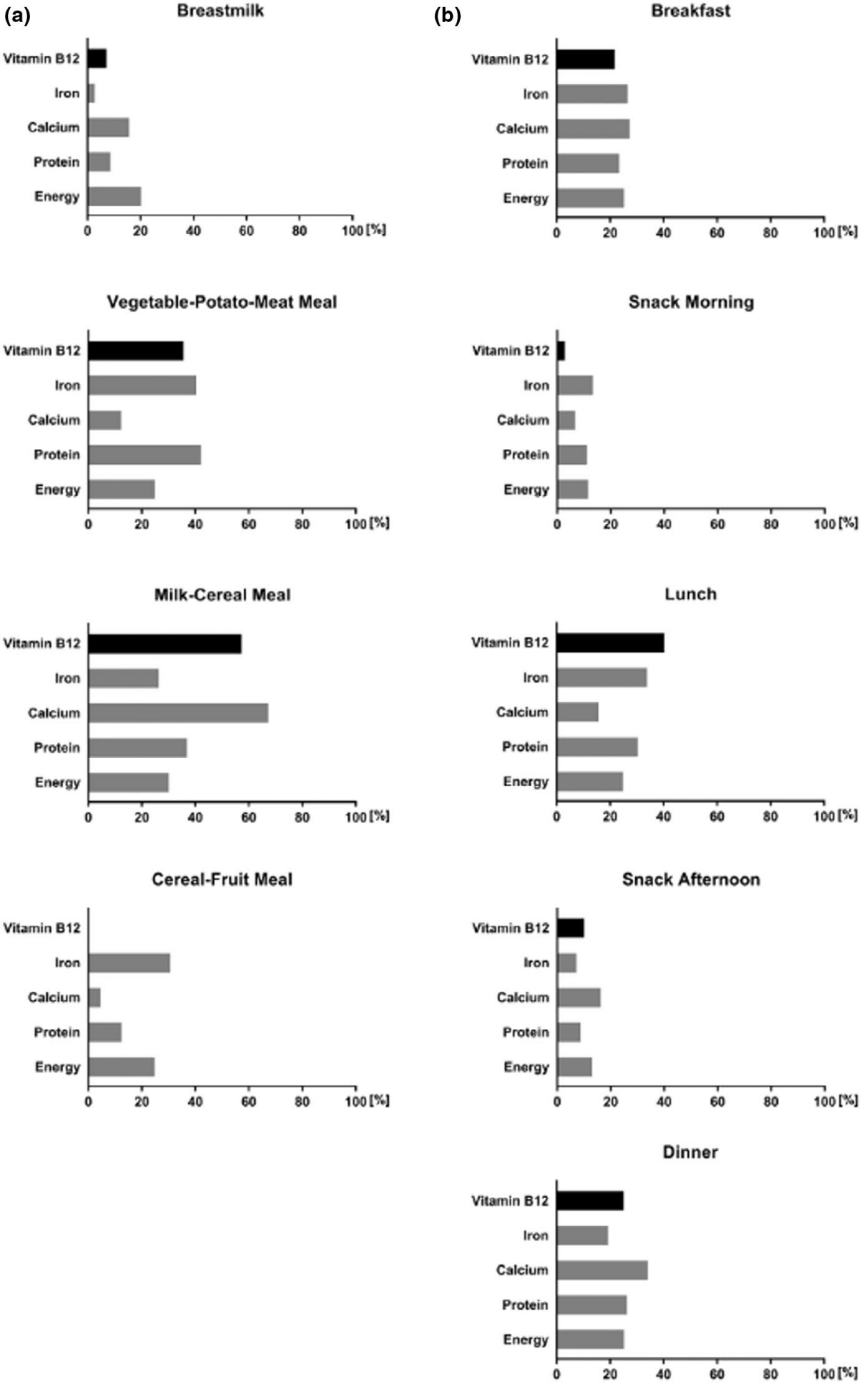
Nutrient profiles of meals of the “Dietary Scheme for the first year of life” (with breastmilk) (a) and of the “Optimized Mixed Diet” for children and adolescents (b) as percentages in the daily intake of energy, Vitamin B12 and other relevant Nutrients.

Among the different meals of the 7‐day menus of the Dietary Scheme (Figure 3a) and the Optimized Mixed Diet (Figure 3b), the milk‐containing meals (Milk–Cereal meal, Breakfast, and Lunch) have high Vitamin B12 densities (nutrient content in relation to energy content) along with high calcium densities, while in the meals containing meat and fish (Vegetable‐Potato‐Meat meal, Lunch) a high Vitamin B12 content is combined with a high iron and protein content. The complementary (vegan) Cereal–Fruit meal and the Snack meals in the Optimized Mixed Diet do not have a relevant role for intake of important nutrients from animal‐based foods (Figure 3).

## 4 DISCUSSION

### 4.1 Planetary health and nutritional adequacy for special populations

In view of the global climate crisis, lifestyle changes are needed, especially in affluent countries. This particularly affects eating habits and requires a paradigm shift in dietary concepts, including the needs of children. While dietary guidelines up to now have focused mainly on meeting nutritional needs to promote health, the climate crisis calls for an extension by considering aspects of the provision of food as well.

Additionally, alternative protein sources are used to substitute animal‐source protein‐rich foods and form an integral part of sustainable food systems to meet the increasing future protein demands. Low‐income populations shift from plant to animal protein sources, while high‐income populations are looking to substitute animal protein sources. (Lurie‐Luke, 2024). And some families may reduce or eliminate animal products from the diets of the children due to various reasons, such as religious beliefs, psychological factors, or financial constraints.

In 2019, the Eat‐Lancet Commission on Healthy Diets from Sustainable Food Systems suggested a global reference diet to improve human health within planetary boundaries (Eat Lancet Commission, 2019). In the typical Western diets, an increase in plant‐based foods at the expense of animal‐based foods is required. However, without professional supervision, traditional plant‐based diets may increase the risk of nutrient deficiencies in vulnerable populations with particularly high growth rates, such as infants and children/adolescents (Moreno et al., 2022). One major problem is the lower than recommended intake of vitamin B12.

### 4.2 Benefits of calculating reduced B12 intake in dietary restrictions

Vitamin B12 deficiency can have various causes and manifest itself in different clinical pictures, ranging from asymptomatic to overt (Van Vlaenderen et al., 2021). Animal foods are the main source of vitamin B12 (Singhal et al., 2017). In addition to some rare congenital conditions, infants with low vitamin B12 intakes, such as infants born to vegan mothers, and children on a vegan diet who do not receive adequate B12 supplementation are particular risk groups for vitamin B12 deficiency (Antony, 2003; Shipton & Thachil, 2015; Stabler, 2013). Especially in the growing age, a sufficient B12 supply should be ensured. For individual counseling of families, it is favorable if the restriction of VB12 intake can be evaluated for different dietary restrictions.

### 4.3 Food sources of vitamin B12 from birth to high‐school age

The German food‐based dietary guidelines cover the whole growth period from infancy to adolescence. They guarantee a balanced nutrient supply without fortified food or supplements and with only moderate amounts of animal‐based foods, especially in the OMD for children from 1 to 18 years of age (Kersting et al., 2017, 2018). Moderation of animal‐based foods already reflects the main recommended approaches toward sustainable dietary habits.

Based on quantitative 7‐day menus according to these guidelines, it was shown that after the early exclusive milk feeding period and the transition first to complementary feeding and later to family food, milk (products) remained by far the most important source of Vitamin B12 in mixed healthy diets, irrespective of age, while meat and fish were of much lower importance and eggs were negligible for adequate Vitamin B12 intake.

Thus, the risk of insufficient vitamin B12 intakes is highest with the exclusion of cow's milk and cow's milk products from the recommended diets (supplementation demand of 60%), followed by the exclusion of meat plus fish (supplementation demand of 30%).

### 4.4 Vitamin B12 intake with milk or supplementation

Milk exclusion is by far the most important risk factor for deficiency of Vitamin B12 in infants and children, even after the milk‐focused infant feeding period (Shipton & Thachil, 2015). Therefore, supplementation of vitamin B12 is required in the absence of milk in the diet, even in basically adequate diets for children. Food alternatives (fish, meat, eggs) are not effective and not practical because nutrient balance would be compromised and menu plans with adequate vitamin B12 intake are hardly feasible without dairy in the family diet.

Irrespective of the reason for a reduction or exclusion of animal‐based foods during the growth period, the extent to which Vitamin B12 supplementation is needed is best decided on a case‐by‐case basis.

### 4.5 Limitations of the study

Our methodological concept and the results of our analysis are a reflection of the dietary habits of infants, children, and adolescents in Germany (Mensink et al., 2017). For a feasible transfer and application, it has to be taken into account that dietary habits and cultures are very different internationally. This is especially true for the introduction of complementary foods in different dietary cultures (Campoy et al., 2018).

Moreover, our analysis does not address aspects of bioavailability and options of reduced intakes of individual types of animal‐based foods (milk or dairy/meat/egg).

## 5 CONCLUSION

Global climate change requires a paradigm shift in diet, especially in affluent countries with a reduction in animal foods. Our analysis shows that the improvement in sustainability comes at the cost of a reduced intake of B12. This is caused by the exclusion of milk from children's diets.

Sustainable nutritional recommendations must therefore seek a balance between planetary health and adequate nutrient supply for children's growth.

## AUTHOR CONTRIBUTIONS

**Hermann Kalhoff:** Conceptualization (lead); formal analysis (equal); investigation (equal); methodology (equal); visualization (equal); writing – original draft (lead); writing – review and editing (lead). **Mathilde Kersting:** Conceptualization (equal); data curation (equal); formal analysis (equal); funding acquisition (equal); investigation (equal); methodology (equal); project administration (supporting); resources (equal); software (equal); supervision (equal); validation (equal); visualization (equal); writing – original draft (lead); writing – review and editing (equal). **Kathrin Sinningen:** Conceptualization (supporting); data curation (equal); formal analysis (supporting); investigation (supporting); methodology (supporting); project administration (supporting); software (supporting); supervision (supporting); validation (equal); visualization (equal); writing – original draft (supporting); writing – review and editing (equal). **Thomas Lücke:** Conceptualization (equal); data curation (supporting); formal analysis (supporting); funding acquisition (lead); investigation (supporting); methodology (supporting); project administration (lead); resources (lead); software (supporting); supervision (equal); validation (supporting); visualization (supporting); writing – original draft (equal); writing – review and editing (equal).

## FUNDING INFORMATION

None.

## CONFLICT OF INTEREST STATEMENT

All authors declare that there are no conflicts of interest.

## ETHICS STATEMENT

Not applicable.

## CONSENT FOR PUBLICATION

All authors of the manuscript have read and agreed to its content and are accountable for all aspects of the accuracy and integrity of the manuscript in accordance with ICMJE criteria. The article is original, has not already been published in a journal, and is not currently under consideration by another journal. All authors agreed to the version of the manuscript to be published.

## Data Availability

The datasets used and/or analyzed during the current study are available from the corresponding author on reasonable request.
